# Supplementary material for: “Pet effect” patterns: Dynamics of animal presence and caregiver affect across (tele)work and non-work contexts
Source: PLoS One. 2025 Feb 20;20(2):e0319009. doi: 10.1371/journal.pone.0319009 (PMC11841913; doi:10.1371/journal.pone.0319009)
Supplement: Attachment — Submitted filename: RR1_Response to Reviewers.docx [file pone.0319009.s001.docx]

# Responses to the editor and the reviewers

First of all, we would like to thank you for giving us the opportunity to revise and resubmit our manuscript. We truly believe that the constructive feedback and suggestions have resulted in an improved version of our manuscript. Below we respond to the different suggestions and comments. We repeat the comments in bold, and reply in italic.

# Editor

**(1) In the online submission form, you indicated that data cannot be shared publicly because of ethical considerations related to the study respondents: the data cannot be made publicly available as per the ethical approval granted for the original studies. Data are available from the Research Ethics Committee (cETO) of the Open University of the Netherlands (OU; U2016/00165/CBO and U2022/08386), (contact via mayke.janssens@ou.nl) for researchers who meet the criteria for access to confidential data. Materials and quantitative analysis methods (including code) that support the findings of this study are available from the corresponding author upon request.**

**All PLOS journals now require all data underlying the findings described in their manuscript to be freely available to other researchers, either 1. In a public repository, 2. Within the manuscript itself, or 3. Uploaded as supplementary information.**

**This policy applies to all data except where public deposition would breach compliance with the protocol approved by your research ethics board. If your data cannot be made publicly available for ethical or legal reasons (e.g., public availability would compromise patient privacy), please explain your reasons on resubmission and your exemption request will be escalated for approval.**

*Thank you for noting this, and our apologies for not expanding further on this issue. We did not explicitly seek participants' consent to publicly share their data, as this was not anticipated at the time of ethical review. Additionally, the data collected involve sensitive, personal information obtained through experience sampling methodology (ESM), in which participants shared details about their daily lives. Given the nature of this data, there are heightened privacy considerations.*

*The policy of the Open University of the Netherlands is to take a cautious approach regarding public data sharing in cases involving such sensitive information. This approach aligns with our commitment to protecting participant confidentiality in studies where data capture highly personal aspects of daily experiences.*

*In light of these considerations, we respectfully request an exemption from the requirement to publicly deposit the data, as we prioritize adherence to the ethical standards set for this study. Access to the data however may be considered for qualified researchers who meet specific criteria, subject to evaluation by the Research Ethics Committee of the Open University (*[*https://www.ou.nl/en/research-ethics-committee-ceto*](https://www.ou.nl/en/research-ethics-committee-ceto)*). The contact person for data availability request is Professor Dr. Nele Jacobs, Department Head, Life Span Psychology, Open University, the Netherlands; e-mail address:* [*nele.jacobs@ou.nl*](mailto:nele.jacobs@ou.nl)*.*

*The collaborating researcher from the Open University (Professor Mayke Janssens), staff of the Open University, will be responsible for all data storage. Data will be stored and preserved for a minimum of 5 years on the internal storing system of the Open University. Data will be removed after this period with at maximum 10 years after publication. Data will not be shared with third parties. E-mail addresses will be kept separate from respondents’ answers and individual data will only be reported at an aggregated level or by means of data suppression. We take all necessary legal precautions regarding data storage and preservation.*

# Reviewer 1

**(1) Thanks for your article which shows a different dimension to human animal interaction. I wondered if you have heard of the below articles or have any thoughts as to how this may modify your discussion and outcome.**

**- Companion animals and child development outcomes: longitudinal and cross-sectional analysis of a UK birth cohort study | BMC Pediatrics | Full Text (biomedcentral.com)**

**-The Complexity of the Human–Animal Bond: Empathy, Attachment and Anthropomorphism in Human–Animal Relationships and Animal Hoarding - PMC (nih.gov)**

**- Working with animals: Implications for employees’ compassion, awe, prosocial behavior, and task performance - Yam - 2023 - Personnel Psychology - Wiley Online Library**

*Thank you for providing these additional perspectives, which may influence our findings. We agree that the studies by Prato-Previde and colleagues (2022) and Yam and colleagues (2023) contribute to our discussion and outcomes, and we have incorporated these references into our manuscript (see page 2, lines 81-85; page 5, lines 144-147; and page 23, lines 600-60). Regarding the study by Purewal and colleagues (2024), we believe it is less directly relevant to our research for three key reasons: (1) their study takes a specific approach to the pet effect in children, while our focus is specifically on contextualizing the pet effect in adults; (2) they assess macro-measures of emotional health (such as self-esteem and anxiety), whereas we are concerned with affective states; and (3) their study applies a macro-perspective across various life stages, which is not aligned with the focus of our research. Therefore, we have not included this paper in the revised manuscript.*

*Purewal, R., Christley, R., Kordas, K., Joinson, C., Meints, K., Gee, N., & Westgarth, C. (2024). Companion animals and child development outcomes: longitudinal and cross-sectional analysis of a UK birth cohort study. BMC pediatrics, 24(1), 578.*

*Prato-Previde, E., Basso Ricci, E., & Colombo, E. S. (2022). The complexity of the human–animal bond: Empathy, attachment and anthropomorphism in human–animal relationships and animal hoarding. Animals, 12(20), 2835.*

*Yam, K. C., Tang, P. M., & Lam, C. (2023). Working with animals: Implications for employees’ compassion, awe, prosocial behavior, and task performance. Personnel Psychology, 76(1), 181-220.*

**(2) Also, whilst I realise that this is a complex topic as well as a complex methodology to navigate around is there a way to optimise pictorials ie showing a diagram of the clusters with linked summary at the end or prior to the discussion to make it easier to follow the assumptions and the outlined associations inferred ? Otherwise well written despite its complexity.**

*We appreciate your recognition of the discussion as well-written despite its complexity. We also agree with your feedback and have addressed it by: (1) improving the resolution of Figure 1; (2) improving the resolution of Figure 2 and adding a content-focused linked summary that highlighting key features of each diagram (see revised Figure 1 and 2, page 16, line 418; and (3) referring to Figure 2 in the Discussion (see page 18, lines 462-463).*

# Reviewer 2

**(1) I have read the manuscript thoroughly and I appreciate the effort. The importance of Human-animal interaction with caregivers cannot be overemphasized and this manuscript lay it bear. I will implore the authors to consider stating years or months the respondent had been with the dog or cat as an inclusion criteria or part of their analysis.**

*Thank you for this valid comment. We agree that the duration of time animals spend with their caregivers may influence our findings. However, we believe our approach—focused on patterns across time and contexts—likely accounts for this factor, at least partially, thereby enhancing the robustness of our methodology. Nonetheless, we have acknowledged the limitation of not measuring cohabitation length in the revised Limitations section (see page 23, lines 600-608).*

**(2) Also, the conclusion was not clearly written so that can be addressed.**

*We apologize for the lack of clarity in the original conclusion and have revised it (see page 21, lines 544-558). We hope the revised version is now more clear.*
